# Supplementary material for: P38 Mediates Tumor Suppression through Reduced Autophagy and Actin Cytoskeleton Changes in NRAS-Mutant Melanoma
Source: Cancers (Basel). 2023 Jan 31;15(3):877. doi: 10.3390/cancers15030877 (PMC9913513; doi:10.3390/cancers15030877)
Supplement: Supplementary file 1 [file cancers-15-00877-s001.zip › cancers-2080355-supplementary.pdf]

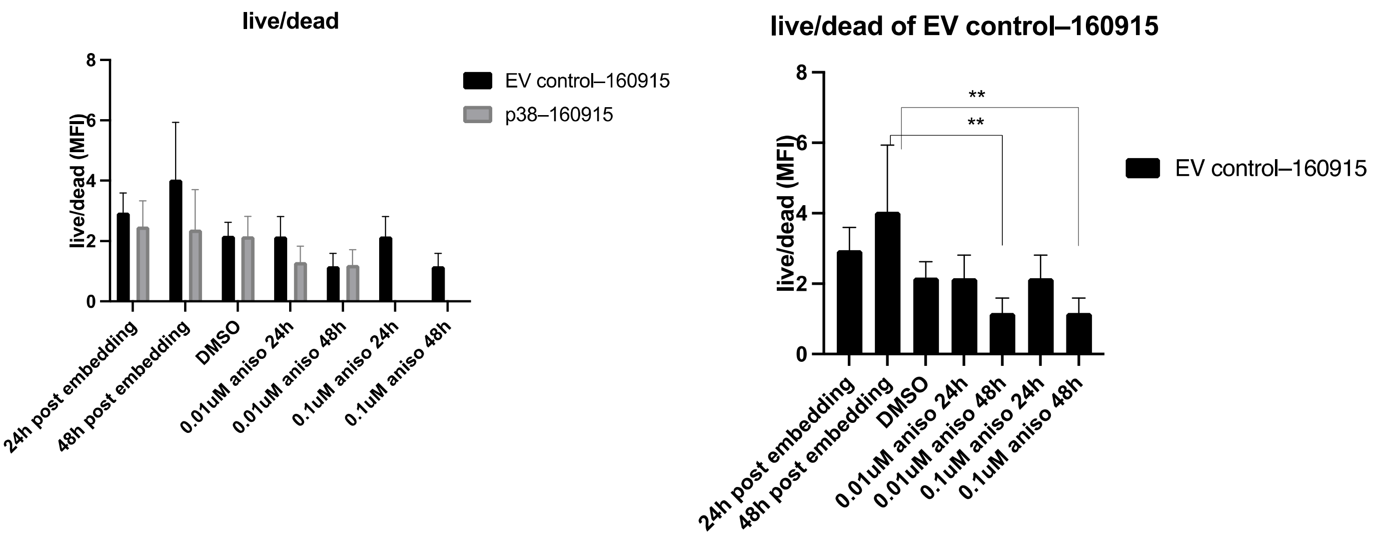

Supplementary Figure S1: Live dead staining of spheroids

A: Ratio of green and red fluorescence intensity to score live/dead cells represented as mean fluorescence intensity (MFI) compared between EV-control-160915 and p38-160915. B: Ratio of green and red fluorescence intensity to score live/dead cells represented as mean fluorescence intensity (MFI) compared between treatment conditions of EVcontrol-160915.

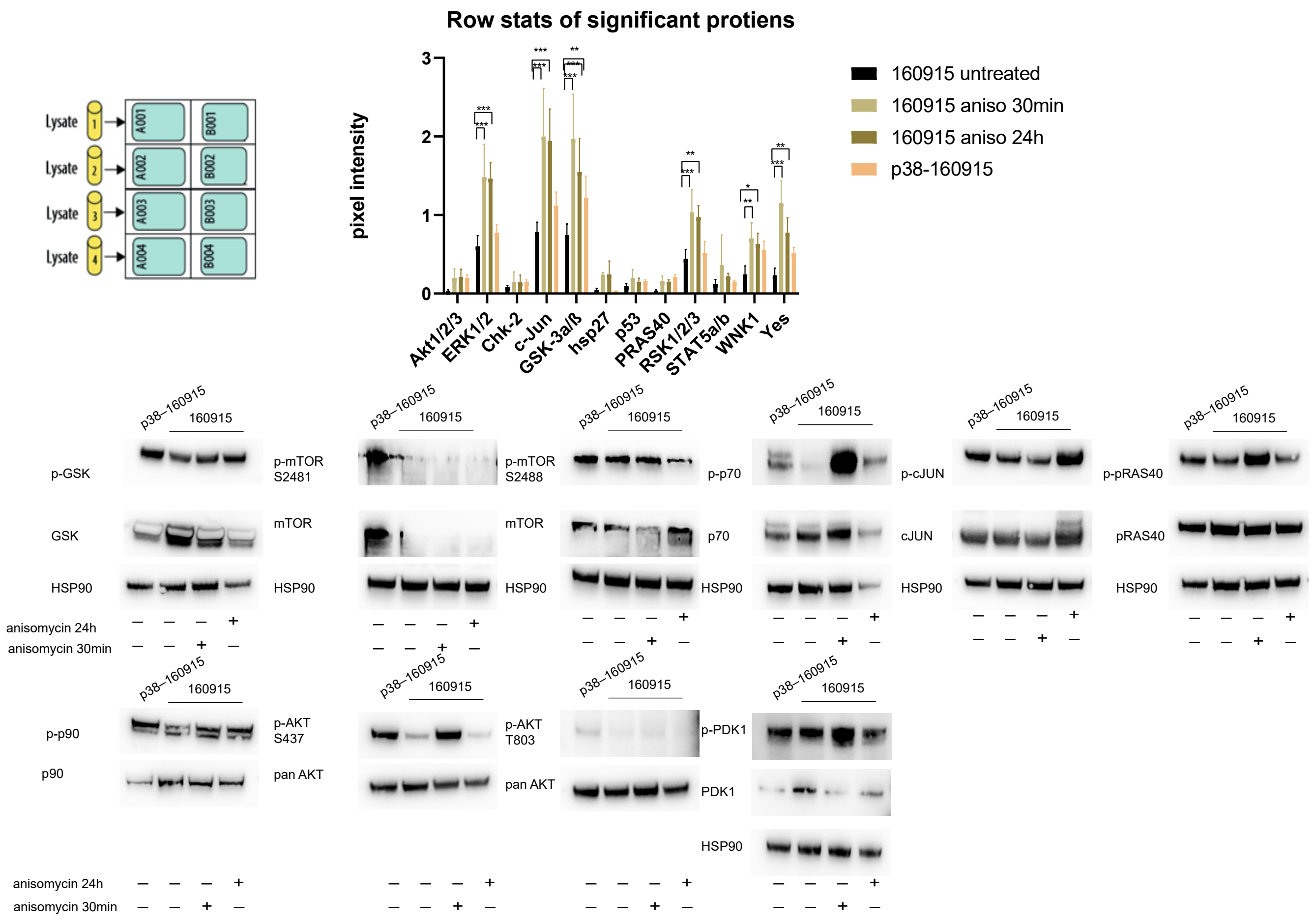

Supplementary Figure S2: Proteome profiler assay

A: Arrangement of membranes (2x) for each sample for a total of 4 samples as provided in the Proteome Profiler Kit. B: Comparison of densitometry analysis of selected proteins between 160915 under untreated, 30min ansiomycin treated, 24h anisomycin treated and untreated p38-160915. C: Protein expression of phospho-GSK, mTORS2481, mTORS2448, p70, cJUN, pRAS40, p90, AKTS437, AKTT803 and PDK1 with HSP90 or total protein as loading control under untreated, 0.1µM anisomycin treatment for 30min and 0.1µM anisomycin treatment for 24h.

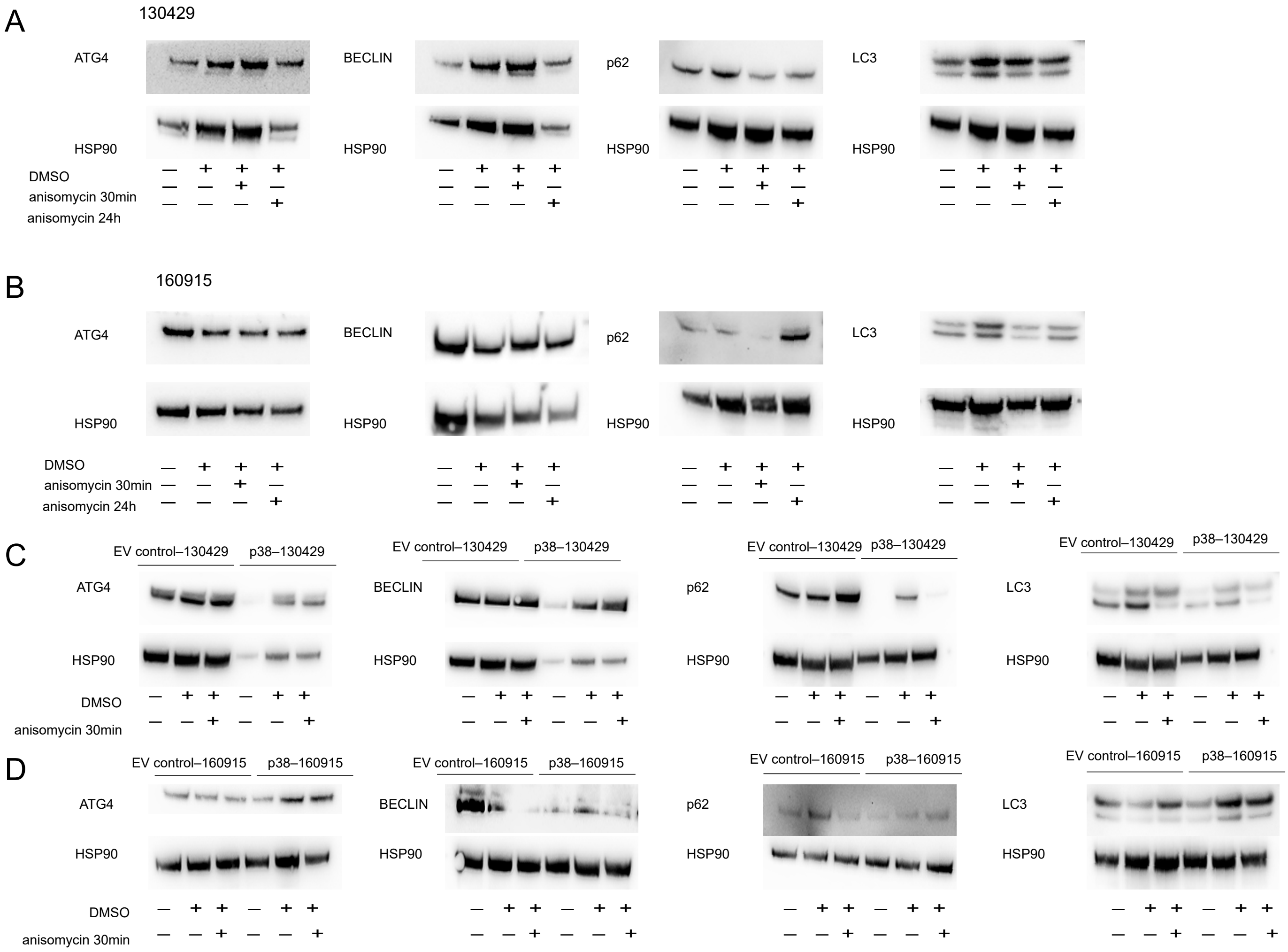

Supplementary Figure S3: Assessing autophagic flux

A: Protein expression of ATG4, BECLIN, p62 and LC3 with HSP90 as loading control under untreated, DMSO treated, 0.1µM anisomycin treatment for 30min or 24h in cell line 130429. B: Protein expression of ATG4, BECLIN, p62 and LC3 with HSP90 as loading control under untreated, DMSO treated, 0.1µM anisomycin treatment for 30min or 24h in cell line 160915. C: Protein expression of ATG4, BECLIN, p62 and LC3 with HSP90 as loading control under untreated, DMSO treated and 0.1µM anisomycin treatment for 30min cell lines EV-control-160915 and p38-160915. D: Protein expression of ATG4, BECLIN, p62 and LC3 with HSP90 as loading control under untreated, DMSO treated and 0.1µM anisomycin treatment for 30min cell lines EV-control-130429 and p38-130429.

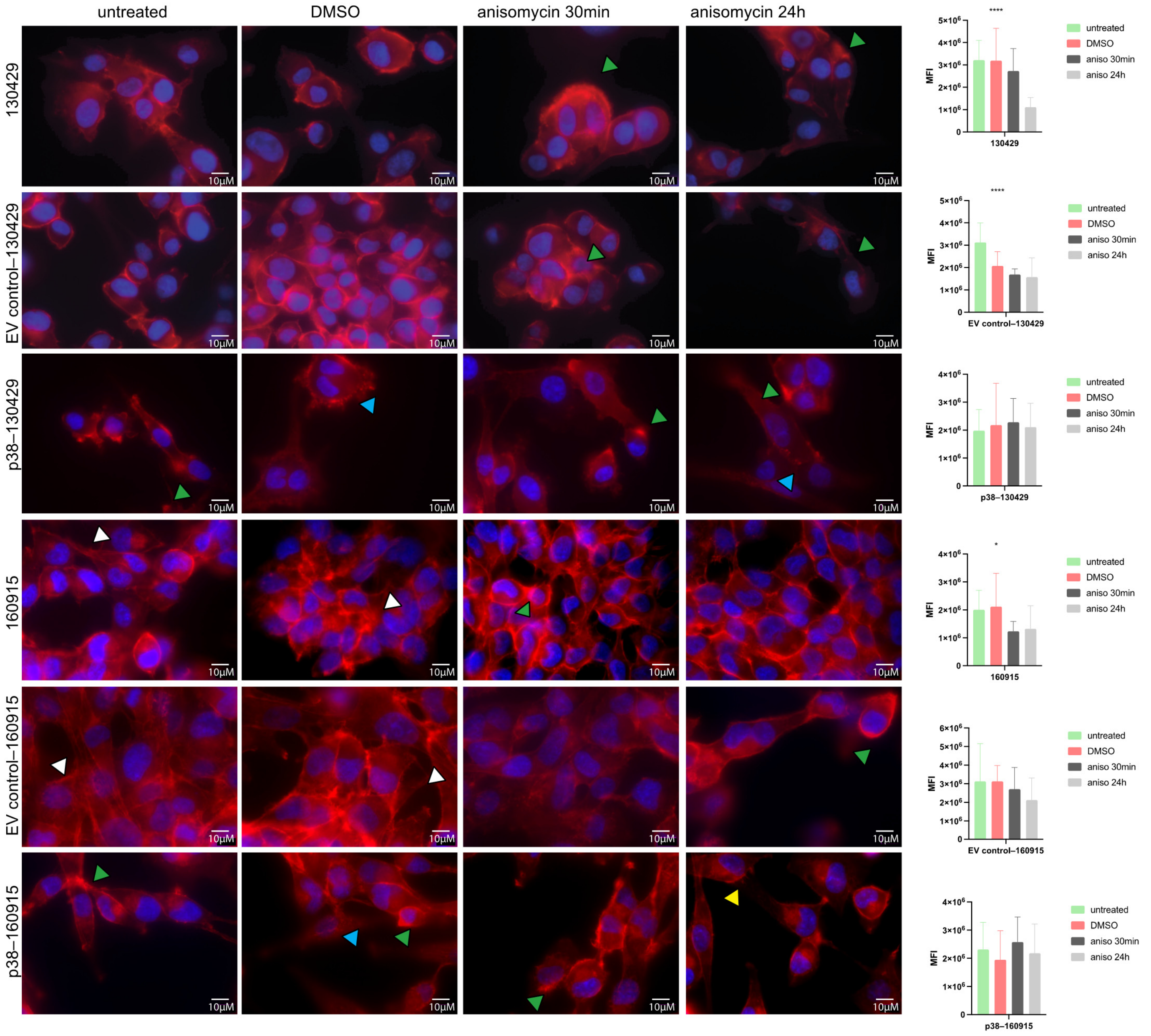

Supplementary Figure S4: Actin depolymerization and reduction in actin MFI with p38 activation

Immunofluorescence staining of SiR-actin (red) and nucleus (blue) in cell lines 130429, 160915, EV-control-130429/160915 and p38-130429/160915 under DMSO or 0.1µM anisomycin for 30min or 24h. Green arrowheads point uncapped, unorganized actin fibers. White arrowheads point to organized perinuclear actin fibers. Yellow arrowheads point to actin fibers breaking off point of contact from one cell to another. Blue arrowhead to point to uneven cytoplasmic deposits. Actin MFI represented as bar graphs.
